# Antibodies Directed against Shiga-Toxin Producing *Escherichia coli* Serotype O103 Type III Secreted Proteins Block Adherence of Heterologous STEC Serotypes to HEp-2 Cells

**DOI:** 10.1371/journal.pone.0139803

**Published:** 2015-10-09

**Authors:** Taseen S. Desin, Hugh G. Townsend, Andrew A. Potter

**Affiliations:** Vaccine & Infectious Disease Organization–International Vaccine Center, University of Saskatchewan, Saskatoon, Saskatchewan, Canada; The Scripps Research Institute and Sorrento Therapeutics, Inc., UNITED STATES

## Abstract

Shiga toxin-producing *Escherichia coli* (STEC) serotype O103 is a zoonotic pathogen that is capable of causing hemorrhagic colitis and hemolytic uremic syndrome (HUS) in humans. The main animal reservoir for STEC is ruminants and hence reducing the levels of this pathogen in cattle could ultimately lower the risk of STEC infection in humans. During the process of infection, STEC_O103_ uses a Type III Secretion System (T3SS) to secrete effector proteins (T3SPs) that result in the formation of attaching and effacing (A/E) lesions. Vaccination of cattle with STEC serotype O157 T3SPs has previously been shown to be effective in reducing shedding of STEC_O157_ in a serotype-specific manner. In this study, we tested the ability of rabbit polyclonal sera against individual STEC_O103_ T3SPs to block adherence of the organism to HEp-2 cells. Our results demonstrate that pooled sera against EspA, EspB, EspF, NleA and Tir significantly lowered the adherence of STEC_O103_ relative to pre-immune sera. Likewise, pooled anti-STEC_O103_ sera were also able to block adherence by STEC_O157_. Vaccination of mice with STEC_O103_ recombinant proteins induced strong IgG antibody responses against EspA, EspB, NleA and Tir but not against EspF. However, the vaccine did not affect fecal shedding of STEC_O103_ compared to the PBS vaccinated group over the duration of the experiment. Cross reactivity studies using sera against STEC_O103_ recombinant proteins revealed a high degree of cross reactivity with STEC_O26_ and STEC_O111_ proteins implying that sera against STEC_O103_ proteins could potentially provide neutralization of attachment to epithelial cells by heterologous STEC serotypes.

## Introduction

Shiga toxin-producing *Escherichia coli* (STEC) is an enteric pathogen that causes diarroheal illness in humans which can lead to hemorrhagic colitis and haemolytic uremic syndrome (HUS), one of the main causes of renal failure in children [1]. Shiga toxins produced by this pathogen play an important role in causing these clinical manifestations. Currently, there is no treatment available for human STEC infections other than supportive care as the administration of antibiotics can exacerbate the disease. STEC O157:H7 is the predominant serotype associated with human infections in North America, while non-O157:H7 serotypes such as O103, O26, O111 are more prevalent in many European countries, South America and parts of Australia [1,2,3] The main reservoir for STEC is ruminants [4] and therefore intervention strategies aimed at lowering the levels of this pathogen in cattle could ultimately result in improved human health [5].

During the process of infection, STEC uses a Type Three Secretion System (T3SS) to inject virulence factors known as effector proteins directly into host cells, leading to the formation of attaching and effacing lesions (A/E) lesions, which are hallmarks of STEC infections. The major structural components of the STEC T3SS include EspA (filament), EspB and EspD (translocon complex) [6]. The STEC T3SS secretes over 50 effector proteins that are encoded on the LEE Pathogenicity Island or elsewhere on the chromosome (non-LEE effectors) [7]. The translocated intimin receptor, Tir, is an effector protein which enters host cells and forms a receptor that binds to intimin that is expressed on the surface of STEC cells [6]. Many studies have shown that the STEC T3SS is essential for colonization of cattle, implying that this is a major virulence factor employed by this pathogen [8,9,10].

Vaccination of cattle with STEC_O157_ T3SP’s has shown to be an effective strategy in reducing the shedding of STEC_O157_ [11,12,13,14,15,16,17,18,19]. However, this protection appears to be serotype specific [20,21]. Therefore, alternative antigens need to be identified that offer protection against non-O157 STEC serotypes. Recently, it has been shown that anti-sera to an extract of STEC_O157_ T3SPs had the highest degree of cross-reactivity with STEC_O103_ recombinant T3SPs [20], suggesting that STEC_O103_ T3SPs may have cross-protective potential. In this study, we tested the effect of sera against STEC_O103_ recombinant proteins on STEC_O103_ and STEC_O157_ adherence to HEp-2 cells. Moreover, we tested the vaccine potential of the recombinant proteins against STEC_O103_ challenge in mice.

## Materials and Methods

### Bacterial strains and growth conditions

The bacterial strains used in this study comprised of *E*. *coli* N01-2454 (O103:H2), EDL933 (O157:H7), CL9 (O26:H11) and CL101 (O111:NM) [22,23]. For cloning and protein expression, we used the *E*. *coli* K-12 lab strains, JM109 (*end*A1, *rec*A1, *gyr*A96, *thi*, *hsd*R17 (r_k_^–^, m_k_^+^), *rel*A1, *sup*E44, Δ (*lac-pro*AB), [F´ *tra*D36, *pro*AB, *laq*I^q^ZΔM15]) and BL21 (F^-^, *dcm*, *ompT*, *hsdS*_*B*_ (r_B_^-^,m_B_^-^), *gal*, λ(DE3)) obtained from Qiagen and Invitrogen, respectively. The strains were grown in Luria Bertani (LB) medium at 37°C in an orbital shaker (250 rpm), unless otherwise stated. *E*. *coli* serotypes O103 and O157 were transformed with a green fluorescent protein expressing plasmid, pNR78, for visualization by flouresence microscopy during the adherence inhibition assays as described [21]. Plasmid pNR78 was constructed in our lab by amplifying the GFP gene from pQBI-25 (Quantum Biotechnologies) which was cloned downstream of the GroEL promoter.

### Protein expression and purification

The STEC serotype O103:H2 T3SS genes *escC*, *espA*, *espB*, *espF*, *espG*, *espR1*, *nleA*, *nleE*, *nleF*, *nleG2*, *nleH*, *sepD*, *tccp2* and *tir* were amplified by PCR (Applied Biosystems) based on the sequence provided by GenBank^®^. Similarly, for cross reactivity studies, *espA*, *espB*, *espF*, *nleA* and *tir* from STEC serotypes O26 and O111 were amplified by PCR. The genes were cloned in either pQE-30 (Qiagen), pET-15b (Novagen), pGEX-5X-1 (GE Healthcare) or pGEX-5X-3, of which the first two are 6x His-tagged protein expression vectors while the latter are Glutathione *S*-transferase (GST)-fusion expression vectors. The constructs were confirmed by PCR and sequencing (Plant Biotechnology Institute, Saskatoon). Proteins were expressed in *Escherichia coli* K-12 lab strains (JM109 or BL21) and purified using either the method described in the QIAexpressionist™ manual (Qiagen) for His-tagged proteins or the GST Gene Fusion System Handbook (GE Healthcare) for the GST-fusion protein. Purified protein samples were greater than 90% pure as determined by SDS-PAGE followed by Coomassie blue staining as described previously [20].

### Raising polyclonal anti-sera to STEC_O103_ T3SS recombinant proteins

Purified recombinant proteins (100 μg each) were formulated with 30% Emulsigen D (MVP Laboratories) and two New Zealand White rabbits (Charles River) per STEC_O103_ recombinant protein were immunized subcutaneously on day 0, followed by booster injections on days 21 and 42. The rabbits were euthanized on day 56 and sera were collected. Antibody titers against STEC_O103_ recombinant proteins were confirmed using ELISA in duplicate wells as previously described [20]. For antibody titer determinations, the cut-off value was considered to be the average of the blank and two standard deviations. All rabbits used in this study were handled and treated in accordance with the guidelines provided by the Canadian Council on Animal Care (CCAC) as administered by the University Committee on Animal Care and Supply (UCACS), protocol 1994–213. This protocol was approved by the UCACS at the University of Saskatchewan for the present study.

### Cell culture

HEp-2 cells (ATCC^®^ CCL-23^™^, CEDARLANE^®^) were grown in HyClone Dulbecco modified Eagle medium (DMEM; Thermo Scientific) supplemented with 10% fetal bovine serum (FBS; PAA Laboratories) and 1% HEPES Buffer (Invitrogen) at 37°C in a 5% CO_2_ incubator. One day prior to the adherence inhibition assays, 10^5^ cells per well were seeded onto eight well chamber slides (Nunc) and allowed to incubate overnight.

### Adherence inhibition assays

**Fig 1:**
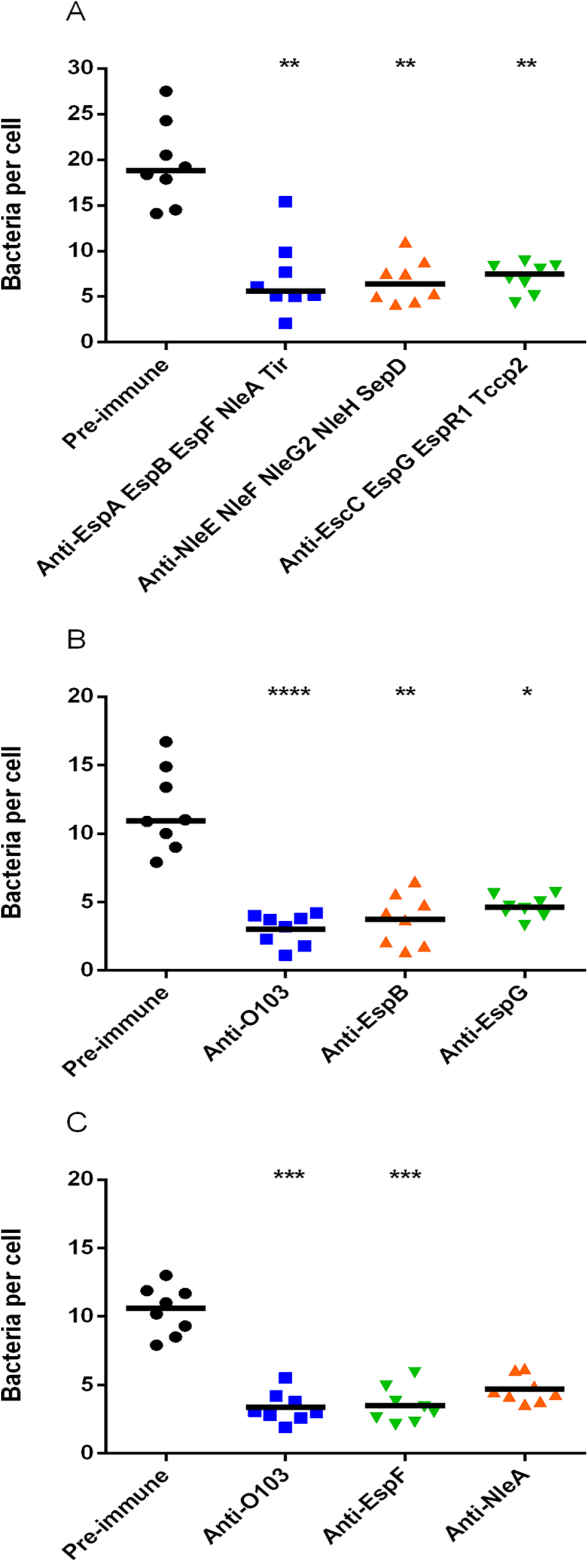
Inhibition of STEC_O103_ adherence to HEp-2 cells by either (A) pooled sera against recombinant STEC_O103_ T3SPs or (B and C) individual serum samples specific for STEC_O103_ recombinant proteins EspB, EspF, EspG or NleA. Anti-O103 refers to antibodies against a secreted fraction of T3SPs from STEC_O103_. Values are expressed as median bacteria per cell from 8 replicates. *, *P* < 0.05; **, *P* < 0.01; ***, *P* < 0.001; ****, *P* < 0.0001.

**Fig 2:**
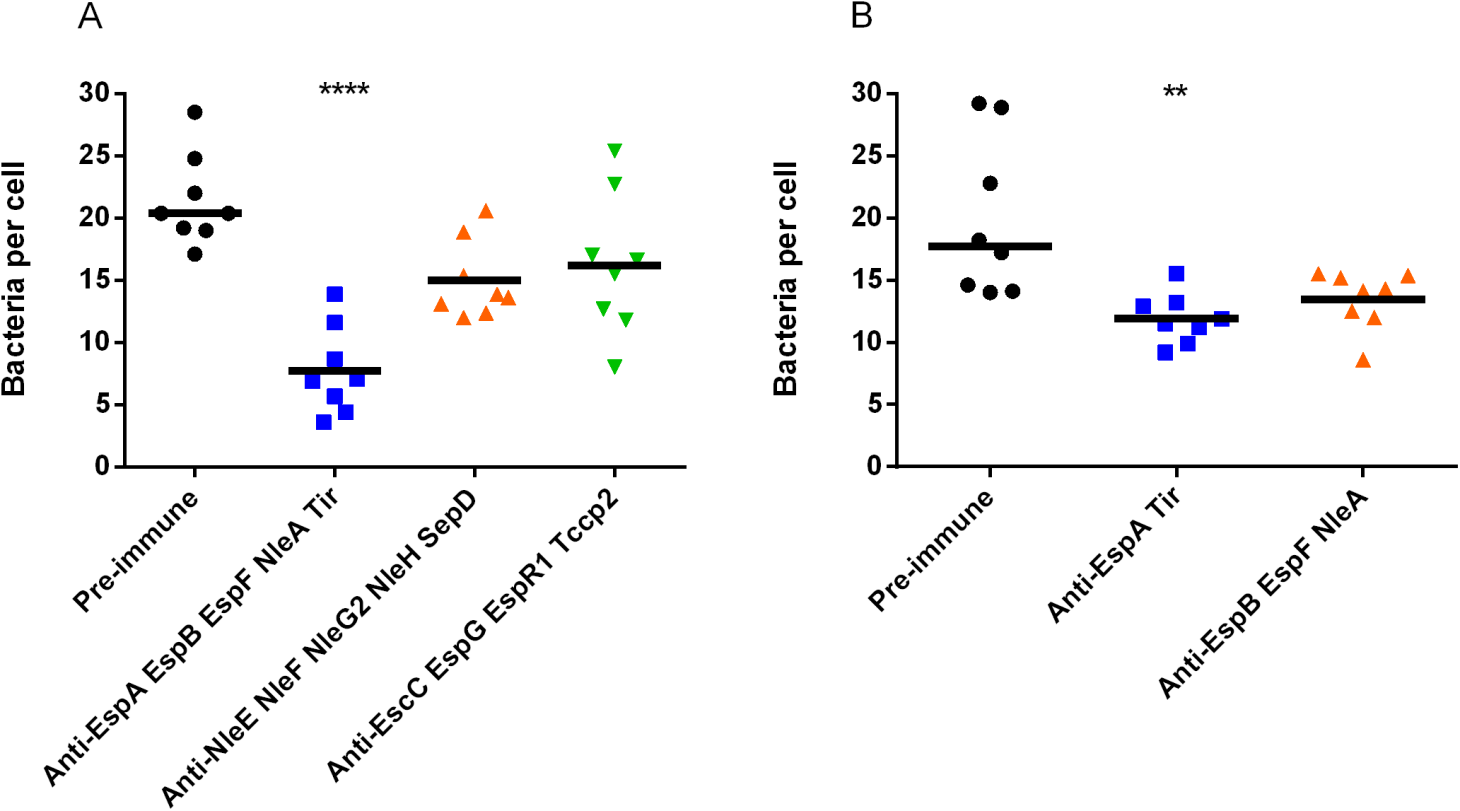
Inhibition of STEC_O157_ adherence to HEp-2 cells by pooled sera against recombinant STEC_O103_ T3SPs. (A) STEC_O157_ adherence to HEp-2 cells was significantly lower in the presence of antibodies against STEC_O103_ EspA, EspB, EspF, NleA and Tir. (B) STEC_O157_ adherence was partially lower in the presence of STEC_O103_ anti-EspA and anti-Tir or anti-EspB, anti-EspF and anti-NleA. Values are expressed as median bacteria per cell from 8 replicates. **, *P* < 0.01; ****, *P* < 0.0001.

Adherence of STEC_O103_ and STEC_O157_ to HEp-2 cells was assessed using an assay described elsewhere [21]. Briefly, an overnight culture of STEC grown in LB media was subcultured (1:100) into DMEM containing 10% FBS and 1% HEPES Buffer for 2 hours (until the OD_600_ was 0.2) at 37°C and 5% CO_2_ without shaking. For testing the effect of pooled sera against STEC_O103_ T3SPs on adherence, HEp-2 cells were infected with 25 μl of STEC (1.7 x 10^6^ colony forming units), 10 μl of each serum and 225 μl fresh DMEM. The effect of individual anti-serum was tested by infecting HEp-2 cells with 25 μl of STEC (1.7 x 10^6^ CFU), 20 μl of anti-serum and 225 μl fresh DMEM (anti-O103 antibodies were prepared as described previously [21]). The chamber slides were incubated at 37°C and 5% CO_2_ for 3 hours (STEC_O157_) or 3.5 hours (STEC_O103_). The slides were washed six times with 200 μl Phosphate Buffered Saline (PBS, 0.1M pH 7.2) and fixed with 200 μl PBS containing 3.7% Formaldehyde. This was followed by two washes with PBS after which the slides were allowed to air dry. Coverslips were mounted with Vectashield^®^ (Vector) containing DAPI and sealed. The slides were visualized under the fluorescent microscope (Axiovert 200 inverted microscope–Zeiss). Bacteria were observed under FITC, while HEp-2 cells were observed under DAPI. Each experimental group was first tested using 2 replicate wells in an 8 well chamber slide and 4 random grids were examined per well under the fluorescent microscope as described below. After observing clear differences in STEC adherence to HEp-2 cells between the different treatments, the experiments were repeated independently on a separate occasion using 8 replicates per test group as previously published [21] with 4 random grids per well used for enumerating the number of STEC per HEp-2 cell. The resulting pictures (4 under FITC and 4 under DAPI) per well (total of 8 pictures per well) were used to enumerate the number of STEC and HEp-2 cells per well. The total numbers of STEC per grid were then divided by the total numbers of HEp-2 cells per grid to determine the number of STEC per HEp-2 cell in one grid. This was repeated for the 8 duplicate wells per group, resulting in a total of 64 pictures per test group. Each data point in Figs 1 and 2 represent the average number of STEC per HEp-2 cell from 4 counts (4 random grids) per well. For statistical analysis, the median STEC per HEp-2 cell across the different test groups were compared using a non-parametric analysis as described below.

### Immunization of mice with STEC_O103_ T3SPs

Twenty four BALB/C mice were obtained from Charles River Canada. Mice were housed at the VIDO Animal Care Facility (University of Saskatchewan) and handled in accordance with the guidelines provided by the CCAC as administered by the UCACS, protocol 1998–0003. This protocol was approved by the UCACS at the University of Saskatchewan for the present study. Mice were randomly divided into two groups with 12 mice per group. The mice were immunized subcutaneously on Day 0 with 100 μl of either PBS (0.1M, pH 7.2) or a pool of STEC_O103_ recombinant proteins EspA, EspB, EspF, NleA and Tir (1 μg of each protein) followed by a second immunization at Day 21. The vaccines were formulated with 30% Emulsigen D (MVP Laboratories). Sera were collected prior to both immunizations on day 0 and day 21 as well as prior to challenge with STEC_O103_ on day 35. Antibody titers were determined using ELISA in duplicate wells as described previously [20]. For antibody titer determinations, the cut-off value was considered to be the average of the blank and two standard deviations. The mice were challenged as described below.

### STEC mouse colonization model

For colonization of mice, we used the streptomycin-treated model as previously described [24,25]. Briefly, mice were given water containing Streptomycin Sulfate (5 g/L) on day 32 for two days. Subsequently, mice were deprived of food and water for 24 hours. On Day 35, mice were orally challenged with 100 μl of 10^9^ cfu of STEC_O103_ Nal^r^ (resuspended in 20% sucrose). The mice were permitted access to food and water containing Streptomycin for the rest of the experiment. Fecal pellets were collected every 3 days for 21 days post challenge. Shedding of STEC_O103_ was monitored by incubating the fecal samples in 1 ml LB broth for 2 hours at room temperature to allow the pellets to soften. The samples were vortexed, serially diluted in PBS and plated on MacConkey Sorbitol Agar containing Nalidixic Acid (15 μg/ml), Cefixime (5 μg/ml) and Potassium Tellurite (2.5 μg/ml). The plates were incubated overnight at 37°C and STEC colonies were enumerated the following day. Bacterial counts were expressed as cfu per gram of fecal content.

### Cross-reactivity of STEC_O103_ T3SS recombinant protein specific sera

Purified STEC_026_ and STEC_0111_ EspA, EspB, EspF, NleA and Tir recombinant proteins were separated by SDS-PAGE and transferred to a nitrocellulose membrane using a Mini Trans-Blot Electrophoretic Cell (Bio-Rad) as per the manufacturer’s instructions. The membranes were probed with either polyclonal sera (1:2500) from mice vaccinated with a pool of the corresponding STEC_O103_ recombinant proteins or with rabbit polyclonal sera (1:2500) raised against STEC_O103_ EspA, EspB, EspF, NleA and Tir. Alkaline phosphatase labeled goat anti-mouse or goat anti-rabbit IgG (KPL) antibodies were used as secondary antibodies (1:2000). The membranes were developed using 5-bromo-4-chloro-3-indolyl phosphate (BCIP) and nitroblue tetrazolium (NBT) salt according to the manufacturer’s instructions (Sigma).

### Statistical analyses

Statistical Analyses were performed using GraphPad Prism 6.02. Adherence inhibition assays were analyzed using a non-parametric analysis (Kruskal-Wallis test) and individual groups were tested using Dunn’s multiple comparison test. Mouse antibody titers were presented as medians plus/minus the 25^th^ and 75^th^ percentile ranges. Differences in immune responses between the vaccine and control groups were tested using non-parametric repeated measures ANOVA. A *P* value of < 0.05 was considered significant.

## Results

### Immune responses against STEC_O103_ recombinant proteins in rabbits

Polyclonal sera were raised against 14 STEC_O103_ recombinant proteins in rabbits in order to test the adherence inhibition effect of the sera in vitro and for cross reactivity studies. All the recombinant proteins induced a significant IgG specific antibody response as determined by ELISA (Table 1). The mean IgG titer across all the proteins was 911,801, while NleE had the lowest antibody titer (151,399) and EspR1 had the highest antibody titer (2,771,000).

**Table 1:** IgG antibody titers specific for STEC_O103_ recombinant proteins in rabbits.

	Pre-immune	Day 56
**Anti-EscC**	87.5	241547
**Anti-EspA**	882.5	838090
**Anti-EspB**	783.5	1256000
**Anti-EspF**	711	618333
**Anti-EspG**	773.5	846100
**Anti-EspR1**	789.5	2771000
**Anti-NleA**	2778	1332000
**Anti-NleE**	555.5	151399
**Anti-NleF**	2748.5	1036000
**Anti-NleG2**	652	1358000
**Anti-NleH**	499.5	614552
**Anti-SepD**	536.5	483176
**Anti-Tccp2**	252.5	402873
**Anti-Tir**	122.5	816244

Antibody titers were determined by setting the cut-off value as the average of the blank and two standard deviations. All serum samples were tested in duplicate wells.

### Antibodies against STEC_O103_ T3SP’s inhibit adherence of STEC_O103_

To test the effect of rabbit polyclonal sera against recombinant STEC_O103_ T3SPs on adherence, we used a functional assay where we measured the level of STEC_O103_ adherence to HEp-2 cells. Our results demonstrate that pooled sera against STEC_O103_ recombinant proteins significantly reduced adherence of STEC_O103_ to HEp-2 cells relative to the group incubated with pre-immune sera (Fig 1A). In order to determine which serum samples were involved in this adherence inhibition effect, we tested specific anti-sera to EspA, EspB, EspF, EspG, EscC, EspR1, NleA, NleE, NleF, NleG2, NleH, SepD, Tir and Tccp2 individually in duplicate. We observed that sera against EspB, EspG, EspF and NleA were involved in blocking adherence (data not shown). To confirm this observation, we performed an adherence inhibition assay where sera against EspB, EspG, EspF and NleA were tested individually with 8 replicates. The data clearly suggest that anti-sera to these four proteins were also highly effective in blocking STEC_O103_ adherence to HEp-2 cells compared to the group treated with pre-immune serum (Fig 1B and 1C).

### Anti- STEC_O103_ T3SP sera have cross-protective potential

In order to determine if antibodies against STEC_O103_ recombinant proteins can block adherence of other STEC serotypes, we evaluated the effect of pooled sera on STEC_O157_ adherence to HEp-2 cells. Interestingly, our results indicate that incubation of STEC_O157_ with anti-sera to STEC_O103_ EspA, EspB, EspF, NleA and Tir significantly lowered adherence to HEp-2 cells, while anti-sera to the other proteins did not have a major effect (Fig 2A). We further investigated this adherence inhibition effect by testing pooled sera against STEC_O103_ EspA and Tir in one group and sera against EspB, EspF and NleA in another group. Adherence of STEC_O157_ to HEp-2 cells was lower in both groups relative to the control group (Fig 2B) but not to the same level as in the pooled group (Fig 2A). These results suggest that antibodies to STEC_O103_ EspA, EspB, EspF, NleA and Tir proteins have a combined effect on blocking STEC_O157_ adherence and that they have cross-protective potential.

### Immunization of mice with T3SP’s from STEC_O103_ induces a strong humoral response but does not affect fecal shedding

**Fig 3:**
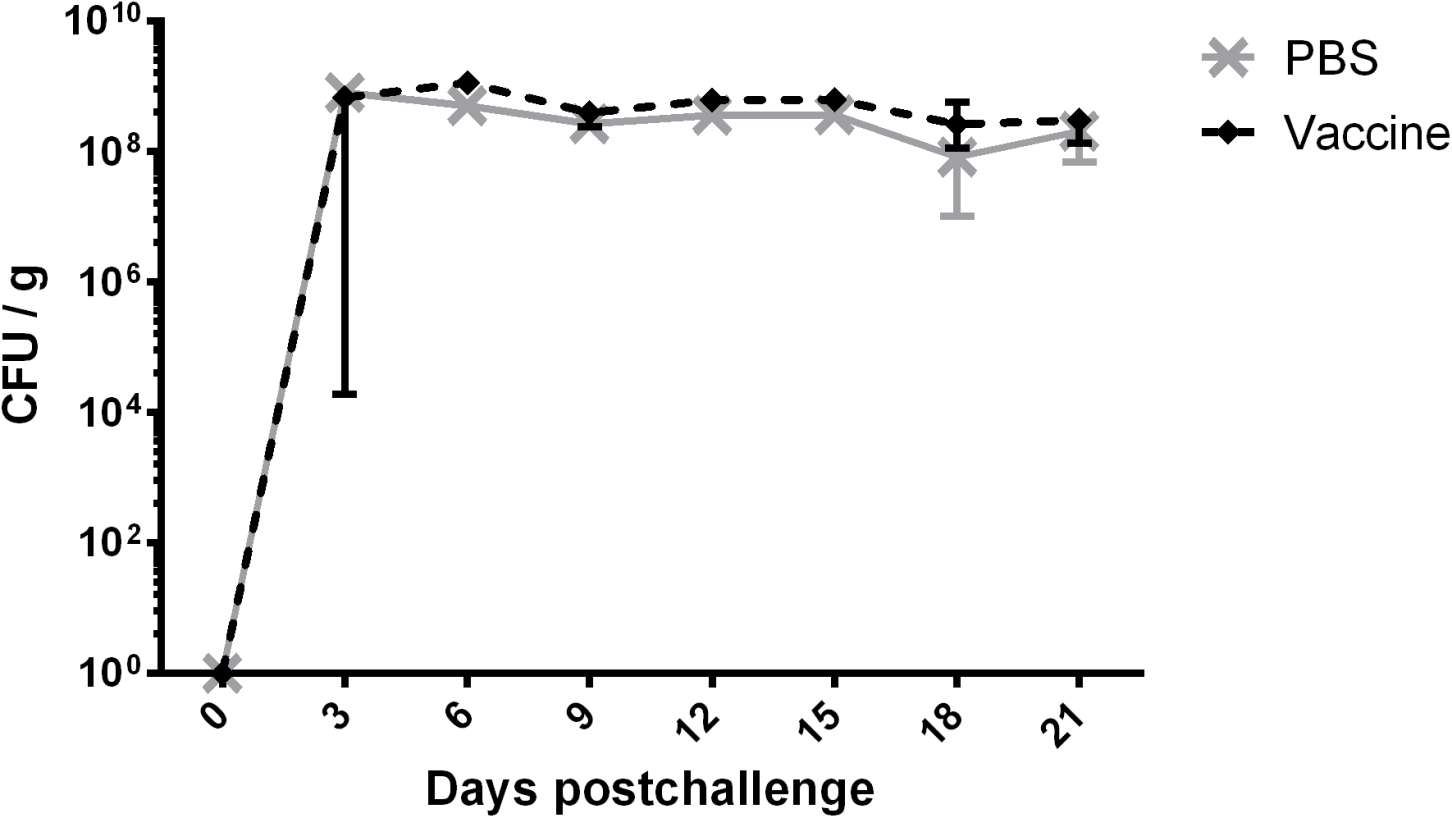
STEC_O103_ shedding in feces following oral challenge in mice. Mice were immunized subcutaneously with either PBS (control) or a pool of STEC_O103_ EspA, EspB, EspF, NleA and Tir followed by a booster immunization three weeks later. Two weeks after the second immunization, mice were orally challenged with 10^9^ cfu of STEC_O103_. N = 12 for both groups. Values are expressed as median cfu per gram of feces.

In order to test the protective capacity of STEC_O103_ effectors, mice were vaccinated subcutaneously with a pool of recombinant proteins and were subsequently infected with STEC_O103_ by oral challenge. Two weeks following the booster immunization, significant EspA-, EspB-, NleA- and Tir–specific IgG titers were detected in the sera relative to the control group (Table 2). In contrast, immunization with EspF elicited a weak IgG specific serum response. To assess the protective capacity of our vaccine, fecal shedding of STEC_O103_ was monitored over 21 days post challenge. The levels of STEC_O103_ were similar in both vaccinates and non-vaccinates throughout the duration of the study, suggesting that antibodies against the antigens used for immunization did not prevent STEC_O103_ from persisting in the intestine (Fig 3), or that the response was not of sufficient magnitude.

**Table 2:** Median IgG antibody titers specific for STEC_O103_ EspA, EspB, EspF, NleA or Tir in mice vaccinated with either PBS or a pool of STEC_O103_ recombinant proteins.

Antibody	PBS				Vaccine				P
	Day 0		Day 35		Day 0		Day 35		
**Anti-EspA**	693	(590–853)	1089	(736–1462)	772	(581–860)	6979	(3109–11618)	0.0007
**Anti-EspB**	235	(157–304)	600	(353–814)	243	(221–477)	294644	(195617–427747)	<0.0001
**Anti-EspF**	882	(742–1240)	1232	(973–5538)	1239	(753–1930)	3822	(1067–13092)	0.052
**Anti-NleA**	481	(161–839)	2734	(422–7809)	552	(250–4314)	1019000	(5364–1385000)	0.0004
**Anti-Tir**	570	(470–1154)	845	(711–3461)	517	(387–652)	280528	(151906–821988)	<0.0001

Values are expressed as median titers. Numbers in parentheses represent the 25^th^– 75^th^ percentile. Antibody titers were determined by setting the cut-off value as the average of the blank and two standard deviations. All serum samples were tested in duplicate wells.

### STEC_O26_ and STEC_O111_ T3SS proteins display significant cross-reactivity with anti-sera to the corresponding STEC_O103_ proteins

**Fig 4:**
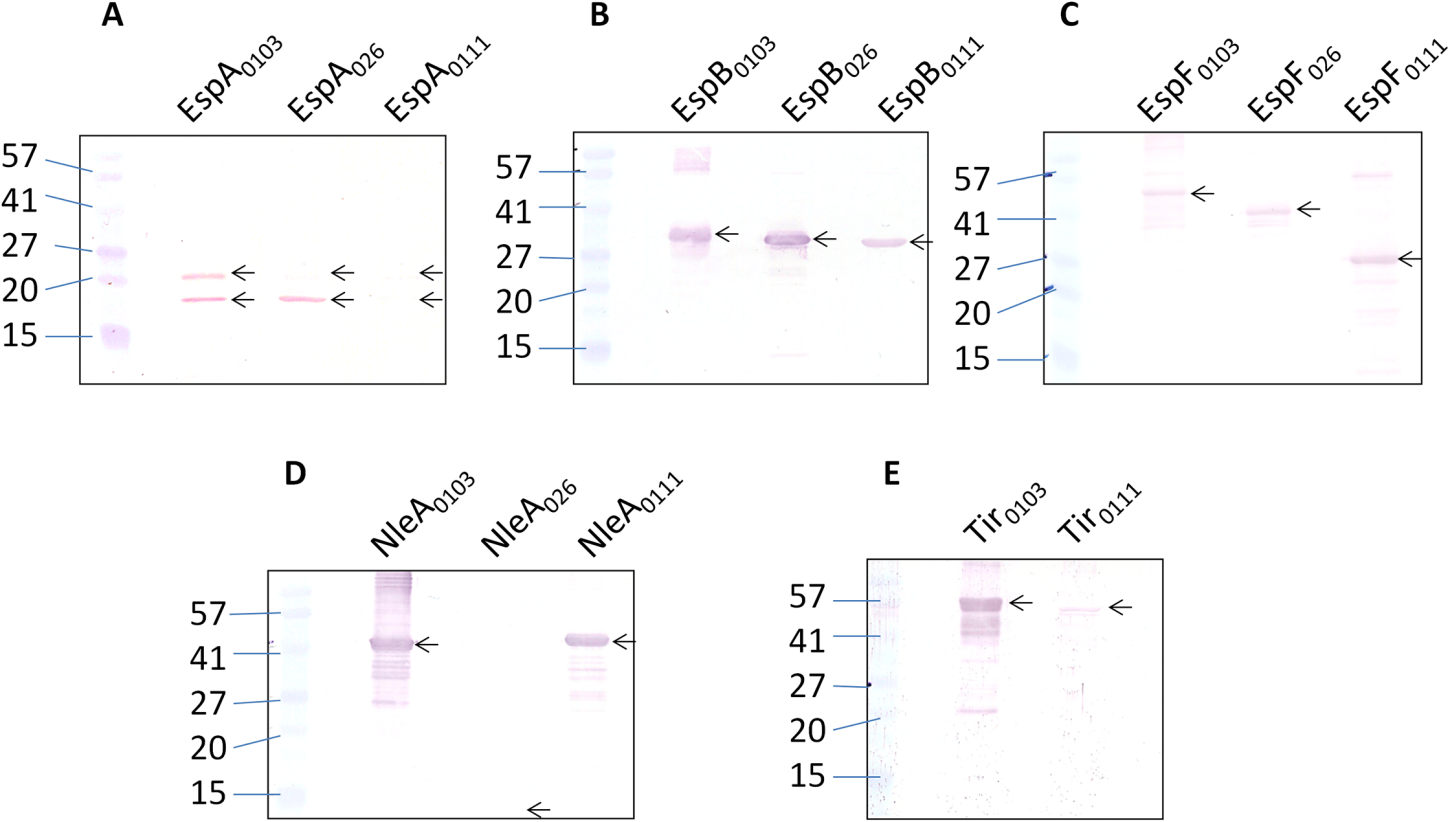
Cross-reactivity of STEC_O103_ EspA, EspB, EspF, NleA and Tir specific rabbit polyclonal sera with the corresponding STEC_O26_ and STEC_O111_ recombinant proteins. (A) Western blot using anti-EspA. Lane 1, marker; Lane 2, EspA_O103_ (20.5 kDa); Lane 3, EspA_O26_ (20.5 kDa); Lane 4, EspA_O111_(20.5 kDa). (B) Western blot using anti-EspB. Lane 1, marker; Lane 2, EspB_O103_ (33.1 kDa); Lane 3, EspB_O26_ (33.2 kDa); Lane 4, EspB_O111_ (32.8 kDa). (C) Western blot using anti-EspF. Lane 1, marker; Lane 2, EspF_O103_ (57 kDa); Lane 3, EspF_O26_ (40 kDa); Lane 4, EspF_O111_ (27 kDa). (D) Western blot using anti-NleA. Lane 1, marker; Lane 2, NleA _O103_ (44 kDa); Lane 3, NleA _O26_ (11.7 kDa); Lane 4, NleA _O111_ (47.5 kDa). (E) Western blot using anti- Tir. Lane 1, marker; Lane 2, Tir _O103_ (56 kDa); Lane 3, Tir _O111_ (56.9 kDa).

**Fig 5:**
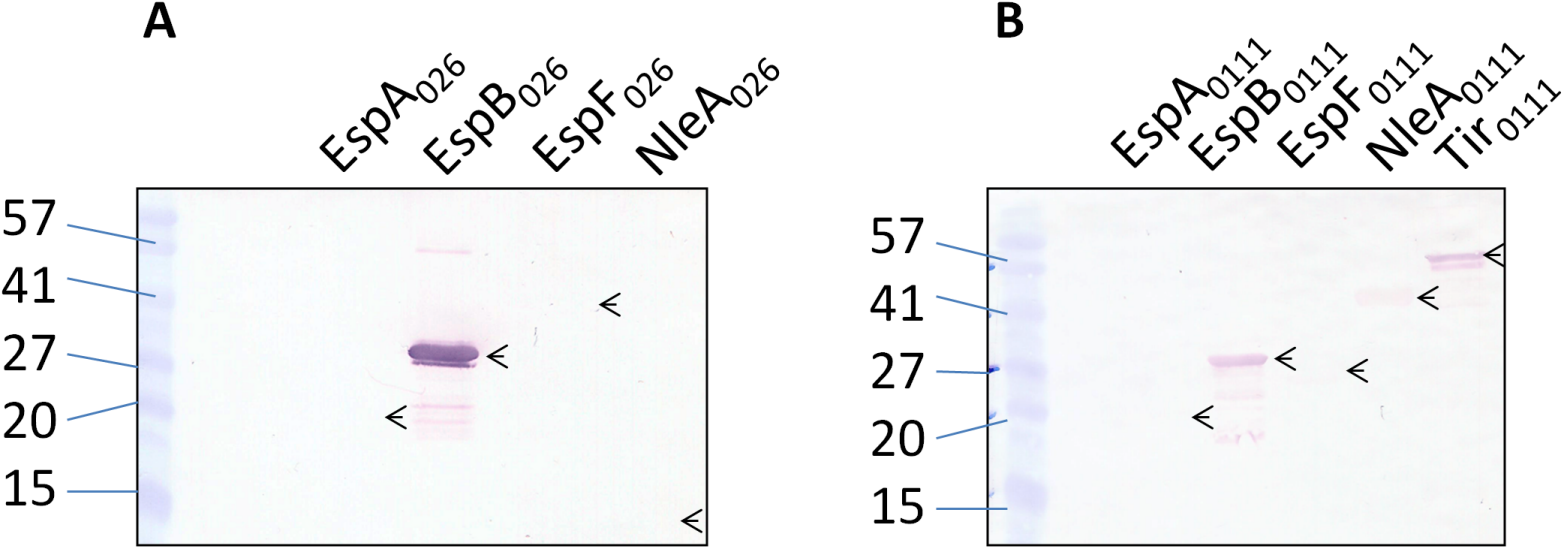
Cross-reactivity of STEC_O103_ EspA, EspB, EspF, NleA and Tir specific mouse polyclonal sera with the corresponding STEC_O26_ and STEC_O111_ recombinant proteins. Western blot using sera from mice immunized with a combination of STEC_O103_ EspA, EspB, EspF, NleA and Tir to test the cross-reactivity with: (A) STEC_O26_ proteins. Lane 1, marker; Lane 2, EspA_O26_ (20.5 kDa); Lane 3, EspB_O26_ (33.2 kDa); Lane 4, EspF_O26_ (40 kDa); and Lane 5, NleA_O26_ (11.7 kDa). (B) STEC_O111_ proteins. Lane 1, marker; Lane 2, EspA_O111_ (20.5 kDa); Lane 3, EspB_O111_ (32.8 kDa); Lane 4, EspF _O111_ (27 kDa); Lane 5, NleA _O111_ (47.5 kDa); and Lane 6, Tir_O111_ (56.9 kDa).

The cross-reactivity of sera against STEC_O103_ T3SS proteins with other STEC serotypes including STEC_O26_ and STEC_O111_, was first tested by western blotting using rabbit polyclonal sera. Our results indicate that EspB_O111_, EspF_O111_ and NleA_O111_ reacted strongly with anti-sera to the corresponding STEC_O103_ proteins, while Tir_O111_ displayed a weaker reaction and EspA_O111_ did not react (Fig 4A). The western blot profile for STEC_O26_ proteins was similar with respect to EspB_O26_ and EspF_O26_. However, EspA_O26_ also reacted strongly, unlike EspA_O111_, while NleA_O26_ did not react (Fig 4B). Subsequently, sera from mice immunized with a pool of STEC_O103_ recombinant proteins was used to study the cross-reactivity with the equivalent STEC_O26_ and STEC_O111_ proteins. The results indicate that EspB_O26_ reacted strongly with the anti-sera while EspA_O26_, EspF_O26_ and NleA_O26_ did not (Fig 5A). In contrast, the western blot profile for the STEC_O111_ recombinant proteins showed a significant degree of cross reactivity for EspB_O111_, NleA_O111_ and Tir_O111_ (Fig 5B). Taken together, the results suggest that EspB_O103_, NleA_O103_, and Tir_O103_ possess significant cross-reactive properties with the corresponding proteins from STEC_O26_ and STEC_O111_. Hence, these proteins may form the basis of a cross-protective vaccine that confers protection against multiple STEC serotypes.

## Discussion

Many efforts have been made to develop STEC_O157_ vaccines using the T3SS proteins as targets in order to reduce the levels of the pathogen in cattle [5,12]. However, these vaccination strategies provide limited protection as they are directed only against STEC_O157_ and they are limited in their benefit [20,21]. Non-O157 STEC serotypes are more prevalent in other parts of the world [1] and with the rise in non-O157 STEC infections in humans [26] as well as the increase in the prevalence of these serotypes in cattle [27], a vaccine that can confer protection against multiple serotypes is more desirable. The aim of this study was to determine if STEC_O103_ T3SS proteins could provide protection against STEC_O103_ as well as other heterologous serotypes using adherence-inhibition assays and the streptomycin-treated mouse model.

We used STEC_O103_ T3SS proteins as targets for a potentially cross protective vaccine since the T3SS proteins encoded by this serotype have previously been shown to have the highest degree of cross reactivity with STEC_O157_, relative to STEC_O26_ and STEC_O111_ [20,21]. Based on this, we over-expressed and purified STEC_O103_ EscC, EspA, EspB, EspF, EspG, EspR1, NleA, NleE, NleF, NleG2, NleH, SepD, Tccp2 and Tir recombinant proteins. In order to test the protective capacity of these proteins, we first examined the effect of rabbit polyclonal sera against the candidate proteins in vitro using a HEp-2 cell adherence inhibition assay which has been successfully used as a functional assay to study STEC adherence [21,28]. This, in turn, may reflect the effect of antibodies on intestinal colonization. Our results demonstrate that pooled sera against STEC_O103_ recombinant proteins significantly inhibited STEC_O103_ adherence. This is in agreement with the findings of Asper *et al*, where anti-sera to all STEC_O103_ secreted proteins blocked adherence by this serotype [21]. We also show for the first time that sera against individual STEC_O103_ recombinant proteins including, EspB, EspF, EspG and NleA, inhibited adherence of the bacteria to HEp-2 cells. Interestingly, pooled anti-sera to STEC_O103_ recombinant proteins EspA, EspB, EspF, NleA and Tir were able to block adherence of STEC_O157_, suggesting that these candidate proteins may provide protection against multiple STEC serotypes. However, it appears that the inhibition of STEC_O157,_ unlike that of STEC_O103_, was due to a combination of STEC_O103_ anti-sera since there was reduced inhibition of STEC_O157_ once the pooled anti-sera were divided into two groups. Taken together, this is the first report which illustrates that sera against STEC_O103_ T3SS recombinant proteins can block adherence of STEC_O157_ to HEp-2 cells.

The streptomycin-treated mouse model [24,29] was used to test the efficacy of the identified candidate STEC_O103_ recombinant proteins as antigens for protection against STEC_O103_. This model was chosen since it has been widely used by various groups to test their STEC vaccines prior to conducting studies in cattle [25,30,31,32,33]. The mice developed strong serum IgG specific titers against EspA_O103_, EspB_O103_, NleA_O103_ and Tir_O103_ following immunization, while the response to the corresponding EspF recombinant protein was weak. The weak response to EspF_O103_ is in line with what was observed for EspF_O157_ in a previous study published by our group [20]. Immunization with STEC_O103_ recombinant proteins did not affect STEC_O103_ fecal shedding over the duration of the experiment relative to the control group. This was unexpected since similar STEC_O157_ based vaccines have been highly effective in mice [30,33]. In addition, a recent vaccination study by our group illustrated that a combination of nine STEC_O157_ recombinant proteins was highly effective in controlling STEC_O157_ fecal shedding in mice (data not shown). It is possible that our STEC_O103_ vaccine may have been more effective against intestinal colonization had it been administered intranasaly. However, both subcutaneous and intranasal immunization of mice with an extract of STEC_O157_ secreted proteins as well as individual recombinant proteins have proven to be highly effective in controlling STEC_O157_ shedding [30]. In addition, the lack of a robust immune response against EspA may have contributed to the persistence of STEC_O103_ in the intestines. Alternatively, since very little work has been done on STEC_O103_ in mice, we speculate that the T3SS may play a different role in STEC_O103_ infection in mice. Therefore, further analysis of the STEC_O103_ T3SS may be required in mice, while a similar vaccine study should be performed in cattle with STEC_O103_.

The serological cross reactivity of STEC_O103_ recombinant proteins EspA, EspB, EspF, NleA and Tir with the corresponding STEC_O26_ and STEC_O111_ proteins was analyzed by western bloting. Overall our results indicate that there was significant cross reactivity of the STEC_O26_ recombinant proteins, EspA_O26_, EspB_O26_ and EspF_O26_ when rabbit polyclonal sera were used. These observations are supported by the protein sequence homology of the STEC_O26_ proteins to STEC_O103_: EspA_O26_ (92%), EspB_O26_ (99%) and EspF_O26_ (91%). The fact that NleA_O26_ did not cross react was not unexpected since the STEC_O26_ genome contains an NleA-like gene which encodes for an 11 kDa protein, while the actual size of NleA_O103_ is 44 kDa. Therefore, sequence homology between NleA_O103_ and NleA_O26_ is expected to be low (58%) with few shared epitopes, if any. We did not show the results for Tir_O26_ since we were unable to express or purify this protein despite numerous attempts. This may be explained by the fact that Tir_O26_ may require co-expression and co-purification with a chaperone [34]. The western blot profile for STEC_O111_ recombinant proteins EspB_O111_, EspF_O111_, NleA_O111_ showed a high degree of cross reactivity with the corresponding sera, while there was lower cross reactivity with Tir_O111_. This is consistent with the observed sequence homologies between the STEC_O103_ and STEC_O111_ proteins: EspB_O111_ (71%), EspF_O111_ (70%), NleA (83%) and Tir (65%). The fact that EspA_O111_ did not react to sera against EspA_O103_ was surprising since EspA_O111_ shares greater than 81% sequence homology to EspA_O103_. The serological cross reactivity of the STEC serotypes O26 and O111 recombinant proteins was remarkably lower when mouse polyclonal sera were used. The difference in the results may be due to differences in recognition of epitopes by the mouse and rabbit immune systems. Overall, the data from both cross reactivity studies suggests that EspB_O103_, NleA_O103_ and Tir_O103_ are highly cross reactive and have the potential to form an efficacious recombinant vaccine that protects cattle not only against STEC_O103_ but other STEC serotypes as well. This finding is supported by two recent studies which demonstrate that EspB_O157_ and Tir_O157_ are immunogenic and protective in cattle against STEC_O157_ [35,36]. Although these studies provide important information about STEC_O157_, this can be used as a basis for conducting similar studies with STEC_O103_ to test for cross serotype protection.

Vaccination with a commercially available STEC_O157_ T3SS vaccine (Econiche^™^) is an effective strategy to control STEC shedding in cattle [37]. Many recent studies have proven that this preslaughter intervention does lead to reduced levels of this pathogen in cattle [38,39]. Moreover, Mathews *et al* have recently predicted that vaccination of cattle against STEC_O157_ will have a significant impact on public health by lowering human STEC infections by 85% [40]. Our in vitro results are the first steps towards a vaccine that may provide protection against multiple STEC serotypes, which is highly desirable for both North America as well as other regions where non-O157 STEC serotypes are more prevalent. The STEC_O157_ SRP^®^ vaccine (contains siderophore and porin proteins) has also shown to be effective in reducing fecal shedding in cattle [41,42]. However, this vaccine also confers limited serotype protection like the Econiche^™^ vaccine [43,44]. Taken together, the need for an STEC vaccine that provides protection against more than one serotype is required and our in vitro results suggest that STEC_O103_ may be a likely candidate, though further testing is required in cattle.
